# Leaf Protein and Mineral Concentrations across the “Miracle Tree” Genus *Moringa*

**DOI:** 10.1371/journal.pone.0159782

**Published:** 2016-07-26

**Authors:** Mark E. Olson, Renuka P. Sankaran, Jed W. Fahey, Michael A. Grusak, David Odee, Wasif Nouman

**Affiliations:** 1 Instituto de Biología, Universidad Nacional Autónoma de México, México, Distrito Federal, Mexico; 2 Department of Biological Sciences, Lehman College, City University of New York, Bronx, New York, United States of America; 3 The Graduate School and University Center-City University of New York, New York, New York, United States of America; 4 Cullman Chemoprotection Center, Department of Medicine, Johns Hopkins University School of Medicine, Baltimore, Maryland, United States of America; 5 United States Department of Agriculture-Agricultural Research Service, Children's Nutrition Research Center, Department of Pediatrics, Baylor College of Medicine, Houston, Texas, United States of America; 6 Biotechnology Laboratory, Kenya Forestry Research Institute, Nairobi, Kenya; 7 Department of Forestry, Range, and Wildlife Management, Bahauddin Zakariya University, Multan, Pakistan; Estación Experimental del Zaidín (CSIC), SPAIN

## Abstract

The moringa tree *Moringa oleifera* is a fast-growing, drought-resistant tree cultivated across the lowland dry tropics worldwide for its nutritious leaves. Despite its nutritious reputation, there has been no systematic survey of the variation in leaf nutritional quality across *M*. *oleifera* grown worldwide, or of the other species of the genus. To guide informed use of moringa, we surveyed protein, macro-, and micro- nutrients across 67 common garden samples of 12 *Moringa* taxa, including 23 samples of *M*. *oleifera*. *Moringa oleifera*, *M*. *concanensis*, *M*. *stenopetala*, an *M*. *concanensis* X *oleifera* hybrid, and *M*. *longituba* were highest in protein, with *M*. *ruspoliana* having the highest calcium levels. A protein-dry leaf mass tradeoff may preclude certain breeding possibilities, e.g. maximally high protein with large leaflets. These findings identify clear priorities and limitations for improved moringa varieties with traits such as high protein, calcium, or ease of preparation.

## Introduction

Protein-energy malnutrition and mineral element deficiencies affect as many as 1 out of 3 people, mostly children in poor countries [1–3], with plant foods being key tools in addressing this situation. In 2011 alone, some 45% of all child deaths involved undernutrition [4]. An urgent global priority is therefore to improve access to healthy food [1]. Most of the world’s poor live in the tropics, and of these the majority live in seasonally dry lowlands [4–7]. Therefore, the development of plants not only with high nutrient levels but also exceptional drought resistance is essential. One of the best-suited plant foods for the dry tropics is the leaf of the moringa tree *Moringa oleifera* (Moringaceae) [8,9].

*Moringa oleifera* is an exceptionally useful multipurpose tree that is probably native to northwestern India but is now cultivated in all tropical countries [10]. It has been used for millennia both medicinally and as a food [11]. It grows very quickly, often exceeding 6 m in height in its first year from seed, and tolerates severe drought and heat, growing optimally in tropical lowlands with high annual low temperatures, low to moderate seasonal rainfall, and well-draining soils [12]. The leaves of the tree contain balanced levels of essential amino acids as well as high levels of protein, calcium, and vitamin A [13–15]. Consequently, the plant is used extensively for low-cost nutrition [9,16,17]. With a range of glucosinolates reflecting its membership in the order Brassicales (the mustard oil plants [18]), all parts of the tree are used medicinally and appear to have potent antioxidant, cancer chemopreventive, and glucoregulatory activity [17,19–21]. The seeds yield a high-oleic oil used in cooking, cosmetics, and as a machinery lubricant [22,23]. After oil extraction, the remaining seed cake can be used to clarify turbid water or to increase protein in animal feed or crop fertilizer [24–26]. Other uses include leaf extract as a leaf-applied fertilizer [27]. Despite the clear utility of the tree, crucial information gaps impede its optimal use in all of these applications, including nutrition.

With regard to nutrition, one of the most significant barriers to optimal use of moringa is a lack of systematic study across the diversity within *M*. *oleifera* as well as the difficulty of comparing across heterogeneous growing conditions such as soil, climate, season, and plant age. In studies of *M*. *oleifera*, reported dried leaf protein values range markedly, from 19.34 to 35.0 g/100g dry weight, and it is not clear what causes this wide variation [8,13–15,28–36]. Macronutrient concentrations also vary across *M*. *oleifera* studies, and as for protein the cause of this variation is not clear. Reported calcium concentrations range from 1440 to 3512.6 mg/100 g of dry leaf [13,15,36,37]. In two studies performed in the same year in Niger, potassium levels varied from 912 to 1770 mg/100g [13,15]. This variability in protein and macronutrient concentrations highlights the need for studies across a wide range of *M*. *oleifera* accessions grown under uniform conditions and processed using uniform laboratory protocols [38].

We address this need by studying plants grown in a common garden including 23 accessions of *M*. *oleifera* reflecting wild type plants and domesticated ones from the Americas, Africa, Asia, and Madagascar. We also include in our study 44 samples from 10 additional *Moringa* species plus an *M*. *concanensis* X *oleifera* hybrid. Most nutritional studies have been centered on *M*. *oleifera* with little exploration of the 12 remaining species in the genus. All the species have local uses [10,11], but there has been little research on the non-*oleifera* species. The only other species for which there is considerable nutritional data is *M*. *stenopetala*. It is consumed as a vegetable as well as used medicinally, especially in central southern Ethiopia [39,40] and the northern Kenyan Rift Valley. *Moringa concanensis* is also consumed to an extent in parts of its range in the Indian subcontinent [41]. Nutritional data are lacking for the remaining species but to guide optimal selection of *Moringa* varieties, a cross-species nutritional survey is essential.

Such a survey requires material from across the range of the genus. *Moringa* species are found naturally in the tropical drylands of Africa, Asia, and Madagascar [42–44]. The well known *M*. *oleifera*, along with *M*. *concanensis*, are native to the Indian subcontinent. The area with the highest number of species is the Horn of Africa, with seven species growing in Kenya, Ethiopia, and Somalia (*M*. *arborea*, *M*. *borziana*, *M*. *longituba*, *M*. *pygmaea*, *M*. *rivae*, *M*. *ruspoliana*, and *M*. *stenopetala*). *Moringa ovalifolia* is found in Namibia and Angola, whereas *M*. *peregrina* grows around the Red Sea, north to the Dead Sea, and around the southern Arabian Peninsula [43]. The two massive pachycaul species *M*. *drouhardii* and *M*. *hildebrandtii* are endemic to Madagascar [44]. This wide range and rarity of some of the species has impeded simultaneous study of multiple species.

We present nutritional data on 11 of the 13 *Moringa* species as well as an *M*. *concanensis* X *oleifera* hybrid. Most of our material comes from our own fieldwork, during which we collected plants in the wild and established them in a common garden. Using this material grown under uniform conditions, we examined crude protein and mineral composition for 67 samples. We identify the species with highest protein and other nutrient contents, highlighting those that offer attractive nutritional or other characteristics. We identify patterns of nutrient covariation that might restrict the possibilities for independently maximizing nutrients such as protein and calcium content under selection, and highlight priorities for further work.

## Materials and Methods

### Plant material and growth conditions

Plants were cultivated in a common garden at the International *Moringa* Germplasm Collection (www.moringaceae.org) near the Chamela Biological Station of the Universidad Nacional Autónoma de México, on the Mexican Pacific coast in Jalisco State. The collection is managed by the first author, and because the garden is expressly for projects such as this, no permission was required. All plants were cultivated and no field work was carried out as part of this project. The area has a tropical monsoonal climate, with a July-October rainy season punctuating a prolonged dry season, though unusual weather conditions brought rain during our sampling January and February 2013–2015. The annual average rainfall is 752 +/- 256 mm, most of which falls in a few large events. Mean annual temperature is 24.9°C, ranging from 14.8 to 32°C [45–47].

The common garden has a uniform base soil consisting of decomposed granodiorites derived from the Vallarta Batholith [48], though given the slightly differing culture requirements of the different species, quantatitve features vary slightly. *Moringa peregrina* and *M*. *ovalifolia* were grown in drier microsites; *M*. *borziana*, *M*. *rivae*, *M*. *ruspoliana*, and *M*. *stenopetala* were grown in intermediate ones, and *M*. *concanensis*, *M*. *drouhardii*, *M*. *hildebrandtii*, *M*. *longituba*, and *M*. *oleifera* were grown at the moistest sites. Thus, the plants were cultivated under identical climatic conditions and in situations maximally similar while still compatible with their slightly differing cultural requirements. The plants were watered every 2 weeks in the dry season and fertilized monthly with a 15:15:15 N:P:K granular fertilizer applied directly to the soil surrounding each plant. Uppermost fully expanded leaves were harvested from randomly selected branches on healthy individuals and air dried for several hours under a fan at ambient temperature before being oven dried to constant weight at approximately 80°C. Constant weight was reached in < 1hr.

### Mineral analyses

Upon transfer to the laboratory, leaflets were separated and the rachises discarded, because it is the leaflets that are usually consumed by people. The exception was *M*. *peregrina*. Because mature leaves of *M*. *peregrina* lack leaflets, we tested the naked leaf rachises (the entire leaf minus the leaflets) separately from the leaflets. The rachis values indicate the levels available for forage, etc., and the leaflet values offer an idea of the values that *M*. *peregrina* would bring to breeding efforts to enhance leaflet nutrient content. Tissues were homogenized with stainless steel grinders. For each sample, 0.25 g of dried tissue was digested in 2 ml nitric acid overnight, then at 100°C for 2 hours. Two ml of 30% H_2_O_2_ was added and the samples were digested for 1 hour at 125°C. A second volume of two ml H_2_O_2_ was added and digested for one hour. The temperature was then increased to 200°C and the samples were evaporated to dryness. Residues were then dissolved in 10 ml 2% nitric acid. The acids used were trace metal grade (Fisher Scientific, Pittsburgh, Pennsylvania, USA) and the water used was deionized via a MilliQ system (Millipore, Billerica, Massachusetts, USA). Samples were analyzed for concentrations of calcium, copper, iron, potassium, magnesium, manganese, molybdenum, nickel, phosphorus, sodium, sulfur, and zinc using inductively coupled plasma-optical emission spectroscopy (CIROS ICP Model FCE12; Spectro, Kleve, Germany) [49].

### Protein

Total protein was measured using the Dumas method in which organic matter is combusted at high temperature and the nitrogen released is trapped and measured as a proxy for protein content [50]. One gram samples of dried leaf powder were sent to the New Jersey Feed Laboratory (Trenton, NJ) for measurement of total protein values (which capture both soluble and insoluble protein). Each sample was run in triplicate. Nine samples were selected randomly, blinded, and resent to the New Jersey Feed Laboratory to check results. In addition, soluble protein was measured in Baltimore at Johns Hopkins University on replicate samples of the same plants by the bicinchoninic acid (BCA) method [51] as adapted for microtiter plates [52]. Whereas soluble protein should in principle always be less than total protein measures, variations recorded by the methods used for each of these determinations have long been the subject of debate [53,54]. We thus employed two separate methods that measure slightly different aspects, but are highly correlated in leaf tissue.

### Leaf Mass per Unit Leaf Area (LMA)

LMA provides an index of how much cell wall material is present in a leaf relative to cell lumen per unit surface area of leaf. Because higher LMA, expressed as dry weight per fresh leaf area, means greater cell wall fraction relative to the cell lumen available for living fraction components such as protein, we expected LMA to predict protein content negatively. We calculated LMA by removing leaflets from the rachises of fresh leaves and measuring leaflet areas with the PC program WINFOLIA (Regent Instruments, Quebec City, Canada). Leaflets were then oven-dried and weighed, with LMA expressed as g/mm^2^ fresh leaflet area.

### Statistical Analyses

We calculated medians per sample, and then medians per species based on them, and used these medians per species for each variable for all analyses. To compare nutrient levels across species, we used Kruskal-Wallis tests after finding that most variables were non-normal and heteroscedastic. We carried out nonparametric posthoc tests using the R package pgirmess [55] to identify homogeneous groups of species, that is, species with non-significant differences in their nutrient levels at α = 0.05.

To examine the degree to which nutrient levels were associated with one another, we calculated Pearson correlations. We standardized variables and carried out a principal component analysis (PCA) to summarize the patterns of covariation between nutrients and to visualize the relative combinations of nutrient levels as described by the first two principal components. Data for the rachis of *M*. *peregrina* were excluded from this analysis, given that we did not have data for its protein content. All analyses were performed in R v. 3.2.1 [56].

## Results

### Ranges of variation

**Fig 1:**
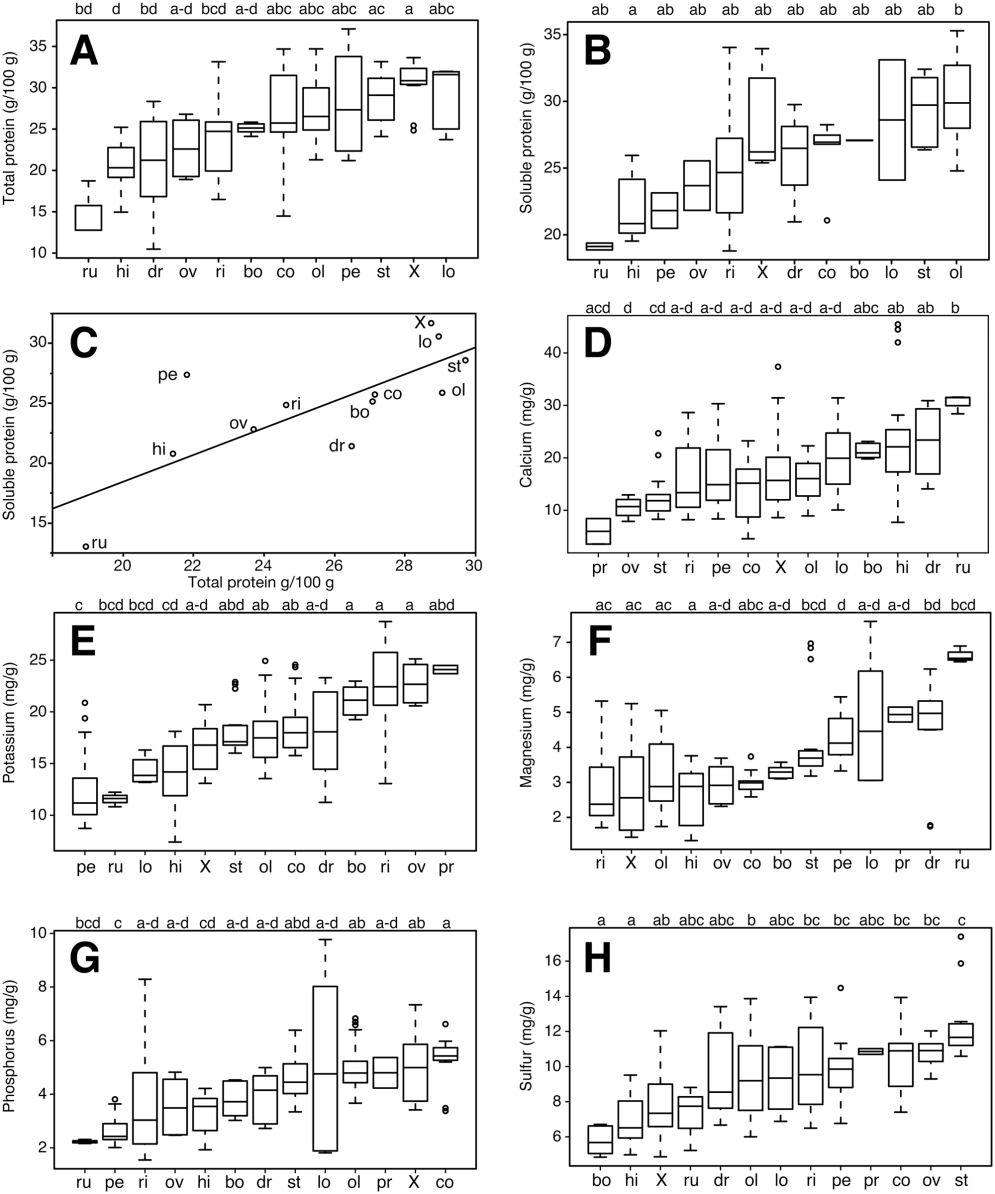
Boxplots and homogeneous groups for protein and macronutrients across *Moringa* species. (A) Total protein, (B) soluble protein, (C) The relationship between soluble and total protein has a slope of ≈1, showing that though there is some variation between methods, this variation seems random and they reflect essentially the same quantities. (D) Ca, (E) K, (F) Mg, (G) P, (F) S. Boxplots are based on the median. Species abbreviations as follows: bo = *M*. *borziana*, co = *M*. *concanensis*, dr = *M*. *drouhardii*, hi = *M*. *hildebrandtii*, lo = *M*. *longituba*, ol = *M*. *oleifera*, ov = *M*. *ovalifolia*, pe = *M*. *peregrina* leaflets, pr = *M*. *peregrina* rachis, ri = *M*. *rivae*, ru = *M*. *ruspoliana*, st = *M*. *stenopetala*, X = *M*. *concanensis* X *oleifera*. Letters denote statistically homogeneous groups as indicated by non-parametric posthoc tests.

**Fig 2:**
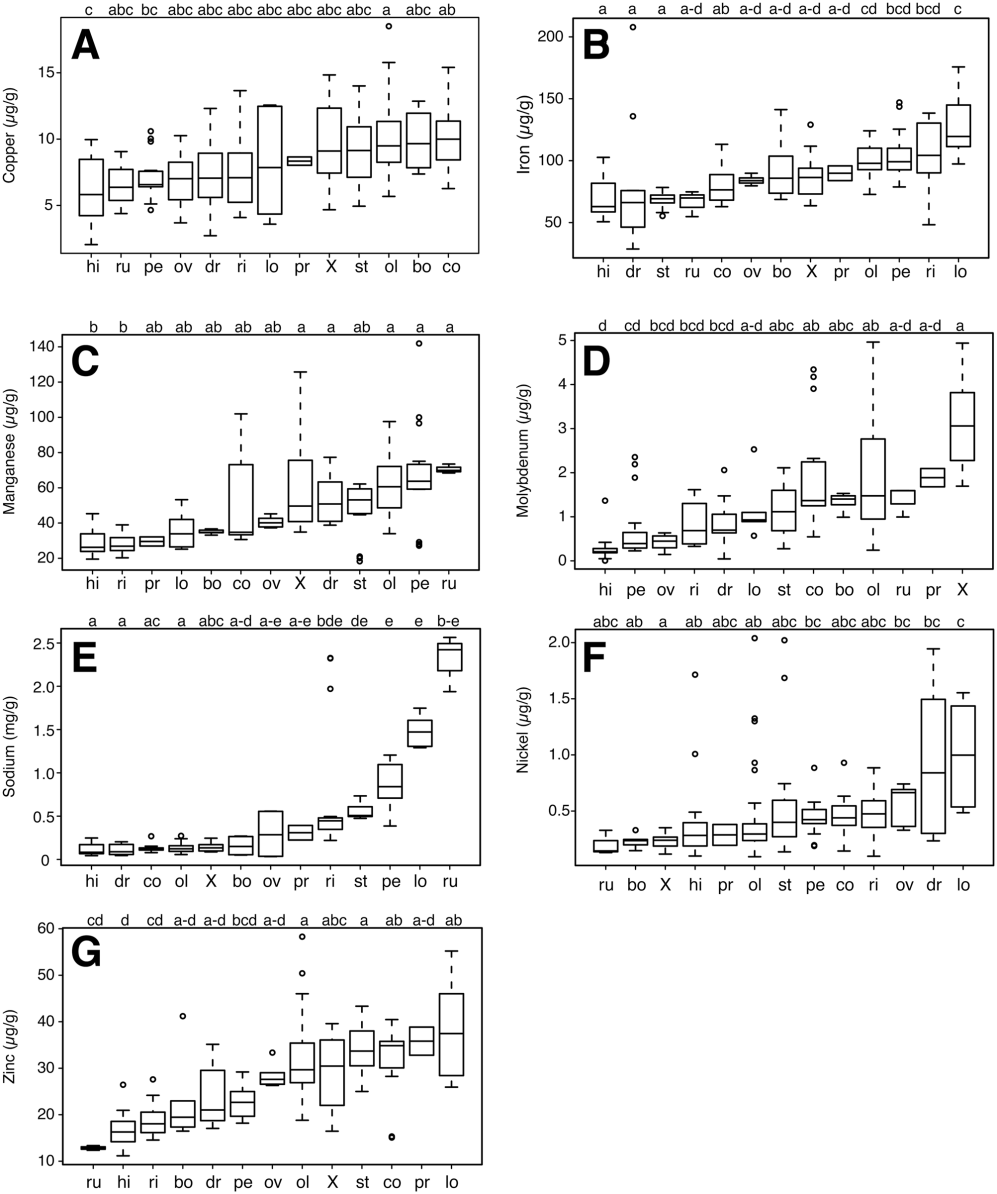
Boxplots and homogeneous groups for micronutrients. (A) Cu, (B) Fe, (C) Mn, (D) Mo, (E) Na, (F) Ni, (G) Zn. Boxplots are based on the median. Abbreviations and conventions as in Fig 1.

Our study examined material from the Americas, Africa, Asia, and Madagascar (S1 Table). Mineral concentrations and protein content in the leaves had markedly different degrees of variation across species. Calculated based on species medians, coefficients of variation were low (< 20%) for copper, iron, sulfur, and both protein measurements (Table 1; raw data in S2 Table). Slightly higher variation (> 20 and < 40%) was observed for practically all macronutrients (calcium, magnesium, phosphorus, and potassium), and for manganese and zinc among the micronutrients. Nickel and molybdenum varied the most, with coefficients of variation > 50%, and for sodium, which had a 30-fold range of variation across species and a coefficient of variation of 129.9%. With regard to these highly varying micronutrients, *M*. *drouhardii*, *M*. *stenopetala*, and *M*. *oleifera* were the species with samples with the highest nickel concentrations, whereas for molybdenum, *M*. *concanensis*, *M*. *oleifera*, and the hybrid between these two species had the accessions with the highest values. For sodium, *M*. *ruspoliana*, *M*. *rivae*, and *M*. *longituba* stood out as the species with the highest concentrations (Table 1). For all nutrients, at least one species differed significantly from the others as suggested by the Kruskal Wallis test (Table 2). Homogeneous groups identified by non-parametric posthoc comparisons for protein and macronutrients are shown in Fig 1 and for micronutrients in Fig 2.

**Table 1:** Leaf nutrient and protein levels, and mass per area, with sample size (N), range “()”, and coefficient of variation (CV) for each nutrient analyzed across *Moringa* species.

	N	Ca (μg/g)	Cu (μg/g)	Fe (μg/g)	K (μg/g)	Mg (μg/g)	Mn (μg/g)	Mo (μg/g)	Na (μg/g)	Ni (μg/g)	P (μg/g)	S (μg/g)	Zn (μg/g)	Total Protein (g/100g)	Soluble Protein (g/100g)	LMA g/mm^2^
*M*. *borziana*	2	21418.9 (19796.5–23125.1)	10.6 (7.4–12.9)	88.7 (68.7–141.3)	21043.5 (19249.4–22971.5)	3270.6 (3100.0–3575.0)	35.3 (33.2–36.7)	1.4 (1.0–1.5)	159.3 (50.1–269.2)	0.2 (0.2–0.3)	3844.9 (3024.3–4532.2)	5834.5 (4845.0–6707.9)	20.2 (16.5–41.2)	27.1 (27.1–27.1)	25.2 (24.1–25.8)	49.79
*M*. *concanensis*	5	15173.4 (4580–23240.5)	10.0 (6.3–15.4)	76.0 (62.9–113.2)	18469.7 (15770.0–24556.7)	2995.0 (2585.0–3738.0)	34.4 (30.7–102.0)	1.4 (0.6–4.3)	115.8 (78.3–267.9)	0.4 (0.14–0.93)	5424.3 (3375.3–6617.4)	10895.0 (7405.0–13932.7)	34.9 (15.1–40.5)	27.2 (21.1–28.3)	25.7 (14.5–34.7)	50.18
*M*. *concanensis* X *oleifera*	4	15880.9 (8615.3–37367.4)	9.7 (4.7–14.8)	87.9 (63.6–129.1)	16782.4 (13084.7–20696.4)	2492.0 (1432.3–5250.3)	49.7 (34.9–125.7)	3.1 (1.7–4.9)	116.1 (86.8–245.0)	0.2 (0.1–0.4)	4961.4 (3415.0–7335.4)	8056.1 (4865.0–12036.2)	30.2 (16.5–39.6)	28.9 (25.4–34)	30.7 (24.8–33.7)	42.93
*M*. *drouhardii*	4	22499.7 (14085.0–30897.0)	7.4 (2.7–12.3)	71.5 (28.7–207.9)	19140.5 (11245.2–23300.0)	4792.1 (1743.4–6238.6)	50.6 (38.8–77.3)	0.8 (0.05–2.1)	80.1 (44.6–203.5)	1.0 (0.2–2.0)	4180.8 (2723.8–4993.7)	9565.4 (6670.0–13411.9)	24.5 (17.1–35.2)	26.5 (21–29.8)	21.2 (10.5–28.4)	58.61
*M*. *hildebrandtii*	7	23583.2 (7720.0–45450.0)	6.3 (2.0–10.0)	61.1 (50.7–195.6)	14704.9 (7410.8–18112.6)	2895.0 (1335.6–3760.0)	25.9 (19.5–45.3)	0.2 (0.01–1.4)	82.9 (43.3–247.2)	0.3 (0.1–1.7)	3730.0 (1928.7–4215.0)	6545.0 (4977.5–9514.1)	16.2 (11.2–26.5)	21.4 (19.5–26)	20.4 (15–25.2)	97.64
*M*. *longituba*	1	19925.3 (10060.0–31431.7)	7.9 (3.6–12.6)	119.5 (97.4–175.7)	13849.7 (13175.0–16308.1)	4459.1 (3053.1–7599.8)	33.9 (25.2–53.3)	0.9 (0.6–2.5)	1472.0 (1290.0–1747.4)	1.0 (0.5–1.6)	4760.1 (1813.0–9772.7)	9344.3 (6880.0–11135.3)	37.5 (25.9–55.2)	28.6 (24.1–33.1)	31.6 (23.7–32.0)	68.82
*M*. *oleifera*	23	16046.7 (8940.0–22284.7)	9.6 (5.7–18.5)	97.9 (72.7–124.1)	17450.0 (13544.2–24930.0)	2833.8 (1738.7–5055.0)	59.8 (34.0–97.6)	1.4 (0.2–5.0)	117.4 (56.0–272.7)	0.3 (0.1–2.0)	4827.4 (3665.0–6829.0)	9363.7 (6001.9–13864.8)	29.1 (18.8–58.3)	29.1 (24.8–35.3)	25.9 (21.3–34.7)	57.60
*M*. *ovalifolia*	2	10543.8 (7895.0–12938.2)	7.4 (3.7–10.3)	84.0 (79.7–89.9)	22729.9 (20578.1–25121.2)	2915.6 (2314.5–3691.3)	40.3 (37.3–45.2)	0.5 (0.2–0.6)	295.6 (33.0–556.7)	0.5 (0.3–0.7)	3521.2 (2467.5–4822.9)	10794.9 (9295.0–12030.6)	27.8 (26.3–33.4)	23.7 (21.8–25.5)	22.7 (18.9–26.8)	66.33
*M*. *peregrina*	6	15041.0 (8358.6–30317.3)	6.6 (4.7–10.6)	102.4 (78.8–147)	10676.4 (8727.1–20875.3)	4047.0 (3326.6–5443.8)	63.7 (27.1–141.9)	0.4 (0.2–2.4)	771.5 (385.8–1205.3)	0.5 (0.2–0.9)	2397.8 (2010.7–3808.8)	9634.2 (6764.6–14473.3)	21.7 (18.2–29.2)	21.8 (20.5–23.1)	27.3 (21.2–37.1)	63.49
*M*. *peregrina* rachis	2	5997.5 (3565.0–8430.0)	8.3 (8.0–8.7)	89.9 (83.9–95.8)	24090.0 (23690.0–24490.0)	4937.5 (4725.0–5150)	29.6 (27.0–32.2)	1.9 (1.7–2.1)	308.4 (224.3–392.5)	0.3 (0.2–0.4)	4800 (4230.0–5370.0)	10857.5 (10695.0–11020.0)	35.8 (32.8–38.9)	-	-	175.75
*M*. *rivae*	5	13374.8 (8215.6–28634.0)	7.8 (4.1–13.7)	91.0 (48.3–138.4)	22311.93 (13062.5–28736.7)	2375.8 (1705.0–5322.7)	26.2 (20.3–39.0)	0.7 (0.3–1.6)	445.8 (219.3–2326.9)	0.5 (0.1–0.9)	3032.9 (1545.6–8290.0)	9536.4 (6494.3–13942.9)	18.1 (14.6–27.6)	24.7 (18.8–34)	24.9 (16.5–33.2)	71.87
*M*. *ruspoliana*	1	31478.6 (28400.0–31571.5)	6.4 (4.4–9.1)	69.9 (54.8–74.8)	11614.6 (10825.7–12230.0)	6545.0 (6446.6–6895.7)	69.9 (68.4–73.5)	1.6 (1.0–1.6)	2422.7 (1938.0–2564.3)	0.2 (0.1–0.3)	2214.8 (2161.5–2311.6)	7743.2 (5225.0–8811.6)	12.8 (12.3–13.4)	19.1 (18.9–19.4)	12.8 (12.8–18.7)	63.42
*M*. *stenopetala*	5	12717.5 (8290.0–24690.0)	9.1 (4.9–14.0)	69.9 (55.4–78.3)	17075.0 (16005.3–22885.0)	3693.6 (3180.0–6968.3)	53.2 (18.3–62.2)	1.1 (0.3–2.1)	507.0 (474.0–733.6)	0.4 (0.1–2.02)	4448.6 (3341.8–6390.0)	12162.0 (10585.2–17395.0)	33.7 (25.0–43.3)	29.7 (26.4–32.4)	28.6 (24.1–33.2)	45.52
CV		38.1	17.7	18.6	23.8	32.5	33.5	65.0	129.9	60.9	25.0	19.5	30.7	13.6	20.6	49.5

Note: The mean value is shown with the range in parentheses. CV for LMA excludes *M*. *peregrina* rachis

**Table 2:** Kruskal-Wallis tests on the 14 nutrients examined.

Nutrient	Kruskal Wallis statistic (χ[2])	Pvalue
Ca	55.69	<0.001
Cu	53.79	<0.001
Fe	92.10	<0.001
K	86.45	<0.001
Mg	64.65	<0.001
Mn	98.25	<0.001
Mo	98.38	<0.001
Na	123.98	<0.001
Ni	56.10	<0.001
P	85.14	<0.001
S	70.82	<0.001
Zn	104.07	<0.001
Soluble Protein	38.32	<0.001
Total Protein	78.05	<0.001

For all tests there were 12 degrees of freedom, except those for protein, for which there were 11 d.f. as a result of excluding the rachis of *M*. *peregrina* from the analyses.

### Protein

Total and soluble protein levels were broadly similar, reflected in the median protein values in Fig 1A–1C. There was marked statistical overlap across most species in their protein levels, but *M*. *hildebrandtii* tended to have the lowest of both protein levels, whereas the highest soluble protein levels were measured in the *M*. *concanensis* X *oleifera* hybrid and *M*. *stenopetala* (Fig 1A), and highest total protein was found in *M*. *oleifera* and *M*. *stenopetala* (Fig 1B). The slope of the OLS regression between the two protein measurement methods was 1.12, very close to 1 (95% CI 0.46–1.78, r^2^ = 0.59, P = 0.004), strongly suggesting that the two methods are reflecting similar results and that the variation between them can be attributed to error (Fig 1C).

### Macronutrients

High macronutrient variation within species translated again into high statistical overlap in homogeneous groups (Fig 1D–1H). However, some trends across species can be noted for each macronutrient. For example, for calcium, *M*. *ovalifolia* and *M*. *stenopetala* tended to have the lowest levels, whereas *M*. *hildebrandtii*, *M*. *drouhardii*, and *M*. *ruspoliana* had the highest (Fig 1D). Regarding potassium, *M*. *ovalifolia*, *M*. *rivae*, and *M*. *borziana* had the highest concentrations, whereas *M*. *ruspoliana* and *M*. *peregrina* tended to have the lowest levels (Fig 1E). In turn, *M*. *rivae*, *M*. *concanensis* X *oleifera*, and *M*. *oleifera* tended to have the lowest concentrations of leaf magnesium, and *M*. *ruspoliana* the highest (Fig 1F). Very strong overlap across species was also observed for phosphorus, with *M*. *ruspoliana* tending to be the species with the lowest levels, and *M*. *concanensis* the highest (Fig 1G). Finally, sulfur levels were lowest in *M*. *borziana* and *M*. *hildebrandtii*, whereas *M*. *stenopetala* tended to be the richest (Fig 1H). As can be observed, trends across species varied widely for the different macronutrients. No species or group of species was rich in all macronutrients.

### Micronutrients

A situation of wide variation and marked overlap across species was also observed for micronutrients. *Moringa hildebrandtii* and *M*. *oleifera* respectively tended to have the lowest and highest levels of copper (Fig 2A), whereas for iron, *M*. *hildebrandtii*, *M*. *drouhardii*, and *M*. *stenopetala* had the lowest and *M*. *longituba* the highest concentrations (Fig 2B). Leaf levels of manganese were lowest in *M*. *hildebrandtii* and *M*. *rivae*, and were highest for a large group of species including *M*. *concanensis* X *oleifera*, *M*. *drouhardii*, *M*. *oleifera*, *M*. *peregrina*, and *M*. *ruspoliana* (Fig 2C). *Moringa hildebrandtii* and *M*. *concanensis* X *oleifera* represented the lowest and highest extremes of molybdenum (Fig 2D). Sodium was one of the few micronutrients with very clear trends. Most species had very low levels, but *M*. *stenopetala*, *M*. *peregrina*, *M*. *longituba*, and *M*. *ruspoliana* tended to have higher concentrations. Two of the samples of *M*. *rivae* also had very high levels of this micronutrient (Fig 2E). Nickel tended to be lowest in *M*. *concanensis* X *oleifera* and highest in *M*. *longituba* (Fig 2F). *Moringa hildebrandtii* tended to have the lowest levels of zinc, whereas with their higher values *M*. *oleifera* and *M*. *stenopetala* tended to form a homogeneous group by themselves with little overlap with other species (Fig 2G). As was the case for macronutrients, no species or group of species tended to be rich in all micronutrients, and many negative as well as positive relationships were observed between variables.

### Relationships between variables

**Fig 3:**
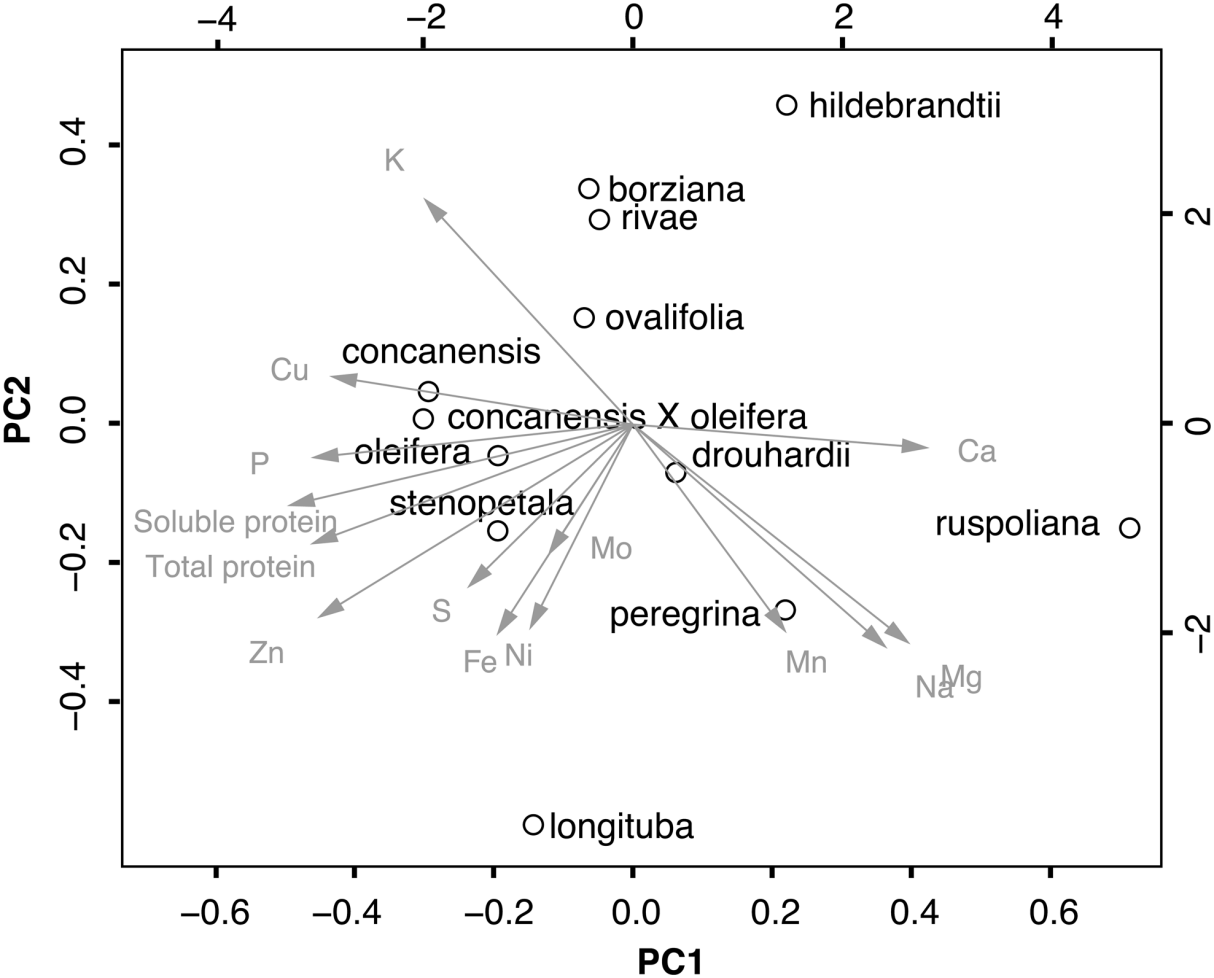
Plot of the first two principal components of the PCA of *Moringa* protein, macro- and micro- nutrient variation across species.

These relationships were manifest in multiple close statistical associations between mineral concentrations and protein content (Table 3). This relatively tight correlation structure meant that in our PCA the first three principal components accounted for 73% of the observed variation in nutrient levels (Table 4). The first principal component accounted for 41%, having very high negative loadings for copper, phosphorous, zinc, and protein, nutrients that were negatively correlated with calcium (Tables 3 and 4, Fig 3). The second principal component, summarizing 17% of the variation, had high negative loadings for iron, magnesium, manganese, sodium, nickel, and zinc, micronutrients that were strongly and mostly positively associated with one another (Table 3), and negatively with potassium, a nutrient that had a positive loading in the second principal component (Table 4, Fig 3). The distribution of species in the PCA plot suggested that *M*. *concanensis* X *oleifera* tended to be rich in phosphorous, copper, protein, and zinc, and poorer in calcium, magnesium, and sodium. *Moringa ruspoliana* had the opposite trend (Fig 3). *Moringa hildebrandtii* tended to have high concentrations of calcium and potassium, but low protein, magnesium, and manganese (Fig 3). *Moringa borziana*, *M*. *ovalifolia*, and *M*. *rivae* had intermediate levels of protein and low levels of nutrients such as iron and magnesium. The remaining species resembled one another in their levels of nutrients (Fig 3). Leaf mass per unit leaf area (LMA) varied from 42.9 g/mm^2^ in *Moringa concanensis* X *oleifera* to 97.6 g/mm^2^ in *M*. *hildebrandtii*. As expected, this index of leaf toughness predicted soluble protein content negatively (Table 3).

**Table 3:** Pearson correlation matrix between nutrients plus LMA.

	Ca	Cu	Fe	K	Mg	Mn	Mo	Na	Ni	P	S	Zn	Soluble Protein	Total Protein	LMA g/mm^2^
Ca	-	0.293	0.324	**0.029**	0.137	0.261	0.817	0.063	0.951	0.191	**0.011**	**0.023**	0.120	**0.019**	0.208
Cu	-0.316	-	0.601	0.153	0.130	0.647	**0.042**	0.105	0.540	**0.010**	0.854	0.086	**0.002**	0.079	0.351
Fe	-0.297	0.160	-	0.845	0.807	0.915	0.853	0.625	0.230	0.719	0.794	0.184	0.308	**0.028**	0.921
K	**-0.603**	0.420	-0.060	-	0.237	**0.035**	0.715	**0.038**	0.978	0.242	0.404	0.388	0.247	0.773	0.238
Mg	0.436	-0.443	-0.075	-0.353	-	0.135	0.902	**0.004**	0.673	0.268	0.954	0.691	0.176	0.073	0.353
Mn	0.336	-0.141	-0.033	-0.588	0.437	-	0.569	0.137	0.665	0.246	0.836	0.533	0.641	0.464	0.146
Mo	-0.071	**0.571**	0.057	0.112	0.038	0.175	-	0.965	0.216	0.113	0.778	0.288	0.156	0.485	0.953
Na	0.529	-0.470	0.150	-0.580	0.741	0.436	-0.013	-	0.935	0.072	0.764	0.409	0.137	0.225	0.887
Ni	-0.019	-0.187	0.358	-0.008	0.130	-0.133	-0.368	0.025	-	0.543	0.267	0.213	0.378	0.341	0.682
P	-0.388	**0.686**	0.111	0.349	-0.332	-0.347	0.461	-0.515	0.186	-	0.336	**0.001**	**0.001**	0.044	0.853
S	**-0.679**	0.057	0.080	0.253	0.018	0.064	-0.087	-0.092	0.333	0.291	-	**0.016**	0.296	0.324	0.698
Zn	**-0.624**	0.495	0.393	0.261	-0.122	-0.190	0.319	-0.251	0.370	**0.819**	**0.651**	-	**0.002**	**0.005**	0.651
Soluble Protein	-0.474	**0.797**	0.321	0.362	-0.418	-0.150	0.436	-0.455	0.280	**0.847**	0.329	**0.801**	-	**0.004**	**0.033**
Total Protein	**-0.664**	0.526	**0.630**	0.093	-0.536	-0.234	0.224	-0.379	0.301	0.588	0.311	**0.752**	**0.767**	-	0.222
LMA g/m[2]	-0.374	-0.282	0.031	0.352	0.281	-0.426	-0.018	-0.044	-0.126	0.057	0.119	0.139	**-0.617**	-0.381	-

Note: N = 13 taxa, except for protein, where N = 12. Correlation coefficients are based on species means and are shown below the diagonal, whereas the significance value is shown above the diagonal. Correlations significant at P<0.05 are shown in bold.

**Table 4:** First three principal components from the PCA and variance explained by each, with nutrients with high loadings in each component shown in bold.

	PC1	PC2	PC3
Ca	**0.314**	-0.040	-0.267
Cu	**-0.312**	0.077	**-0.373**
Fe	-0.140	**-0.342**	0.090
K	-0.221	**0.373**	0.133
Mg	0.298	**-0.366**	-0.074
Mn	0.160	**-0.336**	-0.256
Mo	-0.112	-0.077	**-0.607**
Na	0.277	**-0.374**	-0.066
Ni	-0.106	**-0.328**	**0.401**
P	**-0.342**	-0.057	-0.190
S	-0.175	-0.265	**0.305**
Zn	**-0.337**	**-0.323**	0.011
Soluble Protein	**-0.372**	-0.138	-0.172
Total Protein	**-0.346**	-0.198	0.019
Explained variance	41%	17%	15%
Cumulative explained variance	41%	58%	73%

## Discussion

The nutritional attractiveness of *M*. *oleifera* to dry tropical communities worldwide makes it essential to understand the degree of nutritional variation across *Moringa*. As the first survey to examine this variation across the family, our study shows that no species is high in all or even most nutrients. It highlights wide variation across the genus, with each species (except *M*. *hildebrandtii*) having the highest median value for at least one nutrient (Table 1), with considerable statistical overlap between the ranges across species (Figs 1 and 2). In addition, it points to some potential causes of this variation as well as directions for improvement of cultivated *Moringa* for different applications. We treat these issues briefly here, starting with protein.

### Protein and LMA

*Moringa* is of fundamental interest for tropical malnutrition reduction strategies because of its leaf protein content, and our results help identify the species with the highest and lowest protein levels. Our results confirm *M*. *oleifera*, *M*. *concanensis*, and *M*. *stenopetala* as species with markedly high protein levels (Fig 1A and 1B). *Moringa longituba* had the highest total protein whereas *Moringa ruspoliana* had the lowest protein levels with both methods, though these figures await confirmation because the rarity of both species means that samples from just one individual per species were used for analyses. Even with these considerations, we can safely conclude that a good starting point for the development of even higher-protein moringa would be the two species *M*. *oleifera* and *M*. *stenopetala*

Protein levels were negatively predicted by the amount of mechanical support material in a leaf per unit leaf area, expressed by its leaf mass per unit area (LMA) (Table 1). Across plants generally, LMA is negatively related to leaf lifespan and leaf photosynthetic productivity [57]. In other words, tougher leaves last longer but are less productive photosynthetically per unit time. Plants with higher LMA have greater cell wall fractions as opposed to the cell lumen fractions where photosynthetic and other proteins are located [58]. Photosynthetic productivity in plants is related to the quantity of ribulose-1,5-bisphosphate carboxylase/oxygenase (rubisco). Given that in general roughly half of total leaf nitrogen is accounted for by rubisco plus associated photosynthetic enzymes [59], presumably much of the protein in *Moringa* leaves is accounted for by these proteins. Congruently, LMA in our samples negatively predicted total soluble protein (Table 3). With its conspicuously tough leaflets, *M*. *hildebrandtii* had the highest LMA and one of the lowest total and soluble protein contents. In contrast, high protein species such as *M*. *concanensis*, *M*. *oleifera*, and *M*. *stenopetala* had leaflets with low LMA. Presumably this higher lumen fraction with relatively little cell wall is what permits these species such high protein contents. LMA therefore provides a useful rapid guide for identifying likely high protein variants.

### Macro- and micro- nutrients

The most widely-cultivated species, *M*. *oleifera* and *M*. *stenopetala*, while clearly outstanding with regard to protein, tended to score relatively low in other nutrients. *Moringa stenopetala*, with its consistently high protein levels, tended to fall in statistically low species groupings of all nutrients but sulfur (Table 1, Figs 1 and 2). *Moringa oleifera* tended to fall in groupings with high levels for multiple nutrients, especially calcium, phosphorus, iron, and manganese. This nutritional density along with its cancer chemoprotective and glucoregulatory effects [17] affirm the globally cultivated *M*. *oleifera* as the foremost species for general consumption given current knowledge.

Although *M*. *oleifera* and *M*. *stenopetala* are clear priorities for addressing protein malnutrition globally, the “other” species can potentially also be exploited, and we turn to these now. *Moringa drouhardii*, *M*. *hildebrandtii*, and *M*. *ovalifolia* are conspicious because of their highly drought resistant, massive “bottle tree” habit. Where growth to large size for tapping into relatively deep lenses of soil moisture is possible, these species are clearly outstanding. However, they are also among the least nutritious in general (Fig 3). *Moringa hildebrandtii* was the only species that tended to fall consistently in the lowest statistical groupings for all nutrients. Whereas *M*. *drouhardii* and *M*. *hildebrandtii* at least have the advantage of very fast growth, *M*. *ovalifolia* tends to be much slower, investing for a long time, sometimes years, in underground storage organs before producing a substantial aerial stem, and even then its growth rates are slower. Other African species prove more attractive.

The northeast African closely related species *M*. *borziana*, *M*. *rivae*, *M*. *longituba*, and *M*. *ruspoliana* are of interest for various unique morphological features, as well as for some of their nutrient concentrations. *Moringa ruspoliana* is unique in the family for its leaf morphology. Instead of having large leaves with many tiny leaflets, as does *M*. *oleifera*, *M*. *ruspoliana* has moderate sized leaves that have just 5–7 large leaflets (7 cm or so in diameter). In our experience, one of the most time consuming aspects of the preparation of *M*. *oleifera* leaves for human consumption is the tedious separation of the edible leaflets from the fibrous leaf rachis. Fewer, larger leaflets would reduce preparation time and make moringa much more appealing as a vegetable, as well as reducing production time and cost of moringa leaf powder. Though *M*. *ruspoliana* fell in statistical groupings of species with high calcium, magnesium, manganese, molybdenum, and sodium levels (Figs 1 and 2), it did have among the lowest protein contents in the entire genus. It also had a relatively high LMA. The larger leaflets of *M*. *ruspoliana* require more mechanical support tissue and thus higher LMA [60]. The LMA-protein content tradeoff therefore might present an impediment to developing *Moringa* variants with larger and fewer leaflets. The close relatives *M*. *borziana* and *M*. *rivae* tended to have low nutrient levels. *Moringa borziana* was distinguished only by falling in statistical groupings with high levels of calcium and copper, and *M*. *rivae* in a group with relatively high potassium levels.

*Moringa oleifera* is closely related to two species, *M*. *concanensis* and *M*. *peregrina*, which because of their genetic proximity would be logical first choices for hybrid improvement of *M*. *oleifera*. The *M*. *concanensis* X *oleifera* hybrid tested here was equivalent or in some cases superior to *M*. *oleifera* in protein content as well as in calcium, phosphorus, copper, and molybdenum (Figs 1–3). As the closest living relative to *M*. *oleifera*, it was perhaps not surprising that *M*. *concanensis* tended to resemble *M*. *oleifera* in having high calcium, phosphorus, and protein levels (Fig 1). The other close relative of *M*. *oleifera*, *M*. *peregrina*, tended to have low macronutrient and protein levels in its leaflets. It did however fall in groupings with high levels of iron, manganese, and sodium. These low protein levels again likely reflect the LMA-protein tradeoff. *Moringa peregrina* is unique in that at maturity, the leaves generally drop all or most of their leaflets, leaving naked leaf rachises. Even when present, *M*. *peregrina* leaflets are tiny and with their relatively high LMA (Table 1) are clearly nutritionally inferior to those of *M*. *oleifera*. Interestingly, the leaf rachis of *M*. *peregrina* was, in contrast to the leaflets, in groupings with high levels of potassium, magnesium, sulfur, and molybdenum. Given these nutrient contents in the rachis, *Moringa peregrina* may still be of interest as forage, as well as for its very high seed oil content, but given its tiny, fugacious leaflets it would not seem an attractive prospect for the development of varieties for leaf vegetables. Our results thus provide some indication of which species are most and least promising in *Moringa* breeding, and also suggest which traits are likely to respond independently of others under selection.

The marked associations between traits (Fig 3, Table 3) suggest that selection on some features will inevitably result in concomitant changes in others (Table 4). Selection on, for example, protein levels, will probably alter levels of macro- and micro- nutrients. Calcium, magnesium, and sodium levels, which strongly and positively covaried with one another, covaried to an extent negatively with almost all the remaining nutrients. As a result, selection enhancing protein levels will almost certainly lower calcium levels and vice versa. Fortunately, the levels of calcium in *M*. *oleifera* are sufficiently high as to be nutritionally useful even though its levels were not exceptionally high from the point of view of the variation in the entire genus (Fig 1C; see [61] for a discussion of calcium levels in *M*. *oleifera*). In summary, our results help outline expectations regarding the potential usefulness of the various species as leaf vegetables, as well as which nutrients can likely be maximized independently of others and which will inevitably covary.

### Further Research

Numerous unknowns remain regarding nutritional attributes across *Moringa*. Although we have discussed the potential dietary nutritional value of “other” species, nearly nothing is known regarding features such as the amino acid complement of their proteins, the presence of antinutritional compounds, or even their edibility. Therefore, determining such basic information regarding species of potential interest such as *M*. *longituba* or *M*. *ruspoliana* is a clear priority. The only species known to be eaten as leaf vegetables by local people are *M*. *concanensis*, *M*. *oleifera*, and *M*. *stenopetala*. The other species are all used locally in their native ranges but as medicine, not food. If local tradition is any guide, then it is possible that taste is sufficiently disagreeable or antinutritional factors are sufficiently high in the “other” species as to make them unpalatable.

Moreover, even if a species is edible, very little is known regarding nutrient bioavailability in *Moringa*. Protein is a clear priority in this regard, given that most methods examine total N rather than bioavailable protein. Calcium is another example. Although *M*. *oleifera* is touted for its high calcium levels, anatomical work shows that all plant parts are filled with calcium oxalate crystals [62]. This is potentially important because the calcium in oxalates is not available as a dietary calcium source, and because consumption of high levels of soluble oxalates can contribute to kidney stones [63,64]. Radek and Savage [61] showed that oxalates account for some 38% of total calcium in *M*. *oleifera* leaves. This percentage could seem dismayingly high but the authors found only non-soluble oxalates, which are excreted and thus do not contribute to calculi. Moreover, even though over a third of the calcium was bound up in non-bioavailable oxalates, the high global calcium levels (>20 mg/g of dry leaf in their study, and >35 mg/g in some cases in our data) mean that appreciable potentially bioavailable quantities are nevertheless present. Thus, determining the relationship between palatability and nutrition concentrations across *Moringa* is clearly a priority.

Studies involving higher sample sizes across intraspecific genetic diversity and across different growing conditions are also obvious priorities. Live material for study of most species is extremely hard to come by, and indeed some of the plants in the germplasm collection studied here are the only or are among the very few individuals of their species known in cultivation. The wide range of *Moringa*, across the dry tropics of Africa, Asia, and Madagascar, makes comprehensively surveying the genetic diversity of each species a challenge, as does the remoteness of many of the ranges of the species. There does seem hope for studies with higher sample sizes in the medium to short term, given that in our germplasm collection plants that are currently too small to sample are being continually incorporated into outdoor plantings as they become larger and reach sampling size. Even with these considerations in mind, our results are entirely sufficient to show that the variation across *Moringa* far exceeds that within the currently used species. Moreover, our results help identify species of particular interest, guide expectations regarding which nutrients should covary under selection, and map clear priorities for further work.

## Supporting Information

S1 Table *Moringa* species and samples, authorities, and provenance data. (DOCX)

S2 Table Leaf nutrient and protein levels, and mass per area. (XLS)
