# Supplementary material for: Leaf Protein and Mineral Concentrations across the “Miracle Tree” Genus Moringa
Source: PLoS One. 2016 Jul 26;11(7):e0159782. doi: 10.1371/journal.pone.0159782 (PMC4961408; doi:10.1371/journal.pone.0159782)
Supplement: S1 Table — (DOCX) [file pone.0159782.s001.docx]

**S1 Table. *Moringa* species and samples, authorities, and provenance data.**

| **Sample ID** | **Species** | **Authority** | **Country** | **Locality** |
| --- | --- | --- | --- | --- |
| 1 | *M. borziana* | Mattei | Kenya | not specified |
| 2 | *M. borziana* | Mattei | Kenya | Voi |
| 3 | *M. concanensis* | Nimmo | India | Tamil Nadu, Palni Hills |
| 4 | *M. concanensis* | Nimmo | Pakistan | Jhang |
| 5 | *M. concanensis* | Nimmo | Pakistan | Qalla Didar Singh |
| 6 | *M. concanensis* | Nimmo | Pakistan | Qalla Didar Singh |
| 7 | *M. concanensis* | Nimmo | Pakistan | Qalla Didar Singh |
| 8 | *M. concanensis* X *oleifera* |  | USA | Florida |
| 9 | *M. concanensis* X *oleifera* |  | USA | Florida |
| 10 | *M. concanensis* X *oleifera* |  | USA | Florida |
| 11 | *M. concanensis* X *oleifera* |  | USA | Florida |
| 12 | *M. drouhardii* | Jumelle | Madagascar | Amboasary |
| 13 | *M. drouhardii* | Jumelle | Madagascar | Amboasary |
| 14 | *M. drouhardii* | Jumelle | Madagascar | Amboasary-Berenty area |
| 15 | *M. drouhardii* | Jumelle | Madagascar | seedlot B |
| 16 | *M. hildebrandtii* | Engler | Madagascar | Amboasary-Berenty area |
| 17 | *M. hildebrandtii* | Engler | Madagascar | seedlot C |
| 18 | *M. hildebrandtii* | Engler | Madagascar | seedlot A |
| 19 | *M. hildebrandtii* | Engler | Madagascar | seedlot B |
| 20 | *M. hildebrandtii* | Engler | Madagascar | seedlot B |
| 21 | *M. hildebrandtii* | Engler | Madagascar | seedlot B |
| 22 | *M. hildebrandtii* | Engler | Madagascar | seedlot C |
| 23 | *M. longituba* | Engler | Somalia/Puntland | not specified |
| 24 | *M. oleifera* | Lam. | Thailand | not specified |
| 25 | *M. oleifera* | Lam. | Thailand | not specified |
| 26 | *M. oleifera* | Lam. | USA | not specified |
| 27 | *M. oleifera* | Lam. | India | Tamil Nadu, Chennai, Chrompet |
| 28 | *M. oleifera* | Lam. | Kenya | Isiolo |
| 29 | *M. oleifera* | Lam. | Kenya | Isiolo |
| 30 | *M. oleifera* | Lam. | Kenya | Isiolo |
| 31 | *M. oleifera* | Lam. | Kenya | Isiolo |
| 32 | *M. oleifera* | Lam. | Kenya | Isiolo |
| 33 | *M. oleifera* | Lam. | Kenya | Isiolo |
| 34 | *M. oleifera* | Lam. | Kenya | Isiolo |
| 35 | *M. oleifera* | Lam. | Kenya | Macpalo |
| 36 | *M. oleifera* | Lam. | Madagascar | Tolagnaro |
| 37 | *M. oleifera* | Lam. | Madagascar | Tolagnaro |
| 38 | *M. oleifera* | Lam. | Mexico | El Ranchito, Michoacán |
| 39 | *M. oleifera* | Lam. | Mexico | Mérida, Yucatán |
| 40 | *M. oleifera* | Lam. | Mexico | San Gabriel Chilac, Puebla |
| 41 | *M. oleifera* | Lam. | Mexico | Tuzantla, Michoacán |
| 42 | *M. oleifera* | Lam. | Mexico | Tuzantla, Michoacán |
| 43 | *M. oleifera* | Lam. | Pakistan | Khair Pur |
| 44 | *M. oleifera* | Lam. | Pakistan | Qalla Didar Singh |
| 45 | *M. oleifera* | Lam. | South Africa | not specified |
| 46 | *M. oleifera* | Lam. | Thailand | not specified |
| 47 | *M. ovalifolia* | Dinter and Berger | Namibia | Karibib |
| 48 | *M. ovalifolia* | Dinter and Berger | Namibia | Karibib |
| 49 | *M. peregrina* (2) | (Forssk.) Fiori | Israel | Ein Gedi |
| 50 | *M. peregrina* | (Forssk.) Fiori | Oman | Dhofar |
| 51 | *M. peregrina* (3) | (Forssk.) Fiori | Sudan | not specified |
| 52 | *M. peregrina* | (Forssk.) Fiori | Sudan | not specified |
| 53 | *M. peregrina* | (Forssk.) Fiori | Sudan | not specified |
| 54 | *M. peregrina* | (Forssk.) Fiori | Sudan | not specified |
| 55 | *M. peregrina* rachis (3) | (Forssk.) Fiori | Israel | Ein Gedi |
| 56 | *M. peregrina* rachis (3) | (Forssk.) Fiori | Sudan | not specified |
| 57 | *M. rivae* | Chiov. | Kenya | Marsabit |
| 58 | *M. rivae* | Chiov. | Kenya | Marsabit District |
| 59 | *M. rivae* | Chiov. | Kenya | Marsabit District |
| 60 | *M. rivae* | Chiov. | Kenya | Marsabit District |
| 61 | *M. rivae* | Chiov. | Kenya | Mount Baio |
| 62 | *M. ruspoliana* | Engler | Kenya | Mandera District |
| 63 | *M. stenopetala* | (Baker f.) Cufodontis | Kenya | Parmalok Island |
| 64 | *M. stenopetala* | (Baker f.) Cufodontis | Kenya | Parmalok Island |
| 65 | *M. stenopetala* | (Baker f.) Cufodontis | Kenya | Parmalok Island |
| 66 | *M. stenopetala* | (Baker f.) Cufodontis | Kenya | Parmalok Island |
| 67 | *M. stenopetala* | (Baker f.) Cufodontis | Kenya | Parmalok Island |

When there was little leaf material available per individual, material from more than one individual from the same locality or seedlot was pooled. In these cases, the number of individuals pooled is given in parentheses after the species epithet. Otherwise all accession numbers represent single individuals.
